# Depletion of histone N-terminal-acetyltransferase Naa40 induces p53-independent apoptosis in colorectal cancer cells via the mitochondrial pathway

**DOI:** 10.1007/s10495-015-1207-0

**Published:** 2015-12-14

**Authors:** Demetria Pavlou, Antonis Kirmizis

**Affiliations:** Department of Biological Sciences, University of Cyprus, Nicosia, Cyprus

**Keywords:** Histone N-terminal acetylation, Naa40, NatD, Apoptosis, Colorectal cancer, Epigenetics

## Abstract

Protein N-terminal acetylation is an abundant post-translational modification in eukaryotes implicated in various fundamental cellular and biochemical processes. This modification is catalysed by evolutionarily conserved N-terminal acetyltransferases (NATs) whose deregulation has been linked to cancer development and thus, are emerging as useful diagnostic and therapeutic targets. Naa40 is a highly selective NAT that acetylates the amino-termini of histones H4 and H2A and acts as a sensor of cell growth in yeast. In the present study, we examine the role of Naa40 in cancer cell survival. We demonstrate that depletion of Naa40 in HCT116 and HT-29 colorectal cancer cells decreases cell survival by enhancing apoptosis, whereas Naa40 reduction in non-cancerous mouse embryonic fibroblasts has no effect on cell viability. Specifically, Naa40 knockdown in colon cancer cells activates the mitochondrial caspase-9-mediated apoptotic cascade. Consistent with this, we show that caspase-9 activation is required for the induced apoptosis because treatment of cells with an irreversible caspase-9 inhibitor impedes apoptosis when Naa40 is depleted. Furthermore, the effect of Naa40-depletion on cell-death is mediated through a p53-independent mechanism since p53-null HCT116 cells still undergo apoptosis upon reduction of the acetyltransferase. Altogether, these findings reveal an anti-apoptotic role for Naa40 and exhibit its potential as a therapeutic target in colorectal cancers.

**Electronic supplementary material:**

The online version of this article (doi:10.1007/s10495-015-1207-0) contains supplementary material, which is available to authorized users.

## Introduction

Protein post-translational modifications play a key role in modulating and diversifying protein function [1, 2]. N-terminal acetylation is one of the most common and conserved protein modifications in eukaryotes, occurring in about 80–90 % of all soluble human proteins [3, 4]. For many years, it was believed that the role of this modification was to protect proteins from degradation [5, 6]. However, new findings contradict this conception [7, 8] and further implicate this post-translational modification in the regulation of additional protein properties including enzymatic activity, protein–protein interactions and subcellular localization [9–11]. Importantly, recent studies revealed a role for protein N-terminal acetylation during development of multicellular organisms and proposed that this modification has distinct functions at different developmental stages [12–14]. N-terminal-acetylation, unlike other common modifications, is irreversible and catalysed mainly co-translationally by specific enzymes known as N-terminal acetyltransferases (NATs), which associate with ribosomes [15]. The NAT enzymes transfer an acetyl group from acetyl coenzyme A to the α-amino group of the first amino acid residue of a protein substrate. Six different NAT enzymes have been identified in human cells designated NatA–NatF [16, 17]. Several recent studies have linked the various NAT enzymes to carcinogenesis [18, 19]. For example, Naa10 (the catalytic subunit of NatA complex) has been proposed to behave both as an oncoprotein and as a tumor suppressor. In certain types of cancer tissues like breast, colorectal and lung cancers Naa10 is overexpressed [20–22] and functions as a pro-proliferative and anti-apoptotic factor since its depletion promotes p53-dependent apoptosis. On the other hand, Naa10 expression counter correlates with lung cancer progression and in xenograft experiments Naa10 overexpression suppresses tumor growth and metastasis [23]. Furthermore, NatB is required for cell-cycle progression and depletion of its subunits in cancer cells results in growth arrest and sensitization of cells to pro-apoptotic stimuli [24, 25]. Finally, previous knockdown studies demonstrated that NatC is required for cancer cell survival and proliferation since its depletion induced p53-dependent cell-death [26]. Therefore, manipulating the levels of NAT enzymes in order to induce cell-cycle arrest or apoptosis in cancer cells is considered an attractive therapeutic strategy [18].

Naa40 (also known as NatD, Nat4, or Patt1) is unique among NATs because, unlike the other enzymes that have broad substrate selectivity and acetylate numerous proteins, Naa40 acts as an epigenetic regulator by modifying only histones H4 and H2A [27–29]. Whether additional Naa40 substrates exist remains elusive. However, it was suggested that two other human proteins (H2A.X and SMARCD2) have the proper N-terminal sequence that could be targeted by Naa40 based on its recently determined structure and substrate-specificity [30]. Additionally, four other yeast proteins (Scl1, Ypi1, Dph1 and Lge1) have been proposed as targets of yeast Naa40 [31]. These proteins are typically substrates of NatA, but in yeast lacking NatA activity these proteins remain partially N-terminally acetylated and intriguingly, their N-terminus sequence starts with Ser-Gly, which is compatible with the sequence specificity of Naa40 [32]. Naa40 was first identified and characterised in the yeast *Saccharomyces cerevisiae* [28] and it was later demonstrated that its acetyltransferase activity towards histones is conserved in human cells [32]. This conservation highlights the functional importance of histone N-terminal acetylation. Indeed, we have previously shown that N-terminal acetylation of H4 in yeast promotes ribosomal RNA expression by inhibiting the deposition of an adjacent histone H4 modification, namely arginine 3 asymmetric dimethylation (H4R3me2a) [33]. Furthermore, the activity of Naa40 towards histone H4 at the yeast rDNA region is reduced during calorie restriction suggesting that Naa40 may act as a sensor for cell growth [34, 35]. Consistently, studies in mice showed that liver-specific Naa40 knockout males have aberrant lipid metabolism, reduced fat mass and are protected from age-associated hepatic steatosis [36]. Naa40 deregulation has also been implicated in cancer. In a recent study, Naa40 was shown to be downregulated in hepatocellular carcinoma whereas its overexpression enhanced drug-induced apoptosis that was dependent on its acetyltransferase activity. According to the Human Protein Atlas project, Naa40 protein levels vary in different cancer types, with the highest expression observed in colorectal, ovarian and prostate cancers and the lowest in lymphomas, glioma, renal and liver cancers [37]. The collective data highlight the importance of investigating the role of Naa40 in different cancer tissues.

One of the hallmarks of cancer is the capability of tumour cells to evade programmed cell-death or apoptosis [38]. Normally, apoptosis occurs as a homeostatic and defense mechanism [39] and is mainly induced by two major routes; one that receives signals from the extracellular environment (extrinsic pathway), and another that is triggered by intracellular stimuli (intrinsic or mitochondrial pathway) [40, 41]. The extrinsic pathway is initiated through ligation of cell-membrane death receptors (i.e. the tumor necrosis factor (TNF) receptor superfamily) to their corresponding natural ligands (i.e. FAS), which in turn stimulate the recruitment of the initiator caspase-8 [41]. Upon recruitment, caspase-8 becomes activated and initiates a proteolytic cascade by directly cleaving the downstream effector caspases-3/6/7 [42]. In contrast, the mitochondrial pathway, which is often considered the main barrier to carcinogenesis [38], is initiated by intracellular regulators that belong to the Bcl-2 protein family. This family comprises of anti-apoptotic (like Bcl-2 and Bcl-XL) and pro-apoptotic (like Bax and Bak) factors whose equilibrium determines whether a cell will undergo apoptosis by inducing outer mitochondrial membrane permeabilization (MOMP) [43]. MOMP initially leads to the release of cytochrome-c from the inter-membrane space of the mitochondrion into the cytosol and eventually results in the formation of the apoptosome [44]. The apoptosome mediates activation of initiator caspase-9, which is specific to the intrinsic pathway. Once caspase-9 is activated, it cleaves and activates the executioner caspases-3/6/7. These effector caspases subsequently cleave several other substrates promoting numerous cellular changes that will lead to apoptosis. The established knowledge on the apoptotic pathways is currently being exploited by several therapeutic investigations that are attempting to trigger apoptosis in cancer cells and re-establish this crucial barrier to tumorigenesis [45, 46].

In this study, we sought to explore the link between the histone NAT Naa40 and colorectal carcinogenesis. Initially, we show that depletion of Naa40 in colon cancer cells results in reduced ribosomal RNA expression, which is consistent with the recently described function of yeast Naa40 [33]. We then show that Naa40 is required for the survival of HCT116 and HT-29 colon cancer cells since its depletion induces apoptotic cell-death. In contrast, reduction of Naa40 in mouse embryonic fibroblasts does not affect cell viability. In addition, Naa40-knockdown mediated apoptosis in colon cancer cells is conveyed through the mitochondrial pathway in a p53-independent manner, suggesting that depletion of this enzyme could be a promising therapeutic approach for colorectal cancers irrespective of their p53-status. Altogether, these results highlight the anti-apoptotic role of Naa40 in colorectal carcinogenesis.

## Materials and methods

### Cell culture and reagents

HCT116 cell lines (HCT116 p53+/+ and HCT116 p53−/−) were kindly provided by Dr. Bert Vogelstein (Johns Hopkins University) [47] and were cultured for no more than 15 passages in McCoy’s 5a medium supplemented with 10 % fetal bovine serum and 1 % antibiotic (penicillin/streptomycin). HT-29 and STO cell lines were purchased from ATCC (catalogue no. HTB-38 and CRL-1503, respectively) and were cultured for no more than 10 passages in McCoy’s 5a and DMEM media, respectively, supplemented with 10 % FBS and 1 % pen/strep. The cells were maintained at 37 °C under 5 % CO2. McCoy’s and DMEM media, FBS, penicillin–streptomycin and trypsin were purchased from Gibco, Invitrogen. All cell-lines used were routinely checked for mycoplasma contamination according to the protocol established by Harasawa et al. [48]. The caspase-9 inhibitor z-LEHD-fmk was purchased from BD Pharmingen and DMSO from Gibco, Invitrogen. Target cells were preincubated with 5 µΜ of either z-LEHD-fmk or DMSO, 2 h prior siRNA treatment. Fresh inhibitor was reintroduced to cells 6 and 24 h post-transfection to ensure the continued presence of active inhibitor.

### RNA interference

siRNA against human and mouse Naa40 and negative controls were purchased from GenePharma (Shanghai, China). Human negative control anneals from UUCUCCGAACGUGUCACGUTT and ACGUGACACG UUCGGAGAATT. Human Naa40 siRNA1 and Naa40 siRNA2 sequences were taken from Liu et al. [49], and anneal from CUUUCCCAGUGUUCAAGAATT and UUCUUGAACACUGGGAAAGTT for siRNA1 and GAAGGUUAUGUUAACAGUATT and UACUGUUAACAUAACCUUCTT for siRNA2. siRNA sequences against mouse Naa40 anneal from CAUAAUCAUGGCGCCUAUCTT and GAUAGGCGCCAUGAUUAUGTT for siRNA1 and CAGAGAGUCCUUUCUUGCUTT and AGCAAGAAAGGACUCUCUGTT for siRNA2. Mouse negative siRNA control anneals from CAGCUGAUUUCGUUCGUUCTT and GAACGAACGAAAUCAGCUGTT. All cells were seeded in antibiotic free medium such that they will be ~30 % confluent (HCT116 and HT-29 cells) or ~60 % confluent (STO cells) at the time of transfection and were transfected with siRNA oligo-nucleotides using Lipofectamin RNAiMAX (Invitrogen) according to manufacturer's instructions, to a final concentration of 7.5 nM (HCT116 and STO cells) or 25 nM (HT-29 cells).

### Gene expression analysis

Total RNA was isolated using the RNeasy Mini kit according to the manufacturer’s instructions (Qiagen). DNAse treatment was followed using the TURBO DNA-free kit. To 10 µg of total RNA, 5 µl of TURBO DNAse buffer and 1 µl of TURBO DNAse were added to a final volume of 50 µl and the mixture was incubated at 37 °C for 30 min. The mixture was re-incubated for 30 more minutes after the re-addition of another 1 µl of TURBO DNAse. The reaction ended with the addition of 5 µl of DNase inactivation reagent and the mixture was left to set for 2 min at RT. After centrifugation [2′ × 14000 rpm (18400rcf)], the supernatant was collected in a fresh tube and RNA quantity and quality was measured with nanodrop. Synthesis of cDNA from total RNA was accomplished using PrimeScript RT reagent Kit (Takara). 0.5 µg of total RNA were reversed transcribed in 10 µl of reaction mixture, which included 2 µl of 5× PrimeScript Buffer, 0.5 µl of PrimeScript RT enzyme Mix I, 0.5 µl of oligo dT primer (50 µM), 0.5 µl of random hexamers (100 µM) and RNase free water. The reaction mixture was incubated for 15 min at 37 °C and for 5 s at 85 °C. cDNA was diluted in DNAse RNase-free water so that the concentration of every sample was 0.5 ng/µl. For Real Time PCR, the following mixture was prepared in each well of a multiplate 96-well PCR plate: 5 µl KAPA SYBR Green (SYBR Green Fast qPCR Master Mix), 1 µl cDNA, 3 µl ddH2O and 1 µl primer pair mix (40 ng of each primer). Reactions were incubated in Biorad CFX96 Real-Time System. The PCR products were normalised to those obtained from β-actin mRNA amplification. Primer sequences were designed using Primer 3 and are indicated in Supplementary data 1.

### Western blotting

Cells were washed with ice-cold PBS (1×) and were solubilised in Lysis buffer composed of 50 mM Tris–Cl (pH 8), 3 mM EDTA, 100 mM NaCl, 1 % Triton-X-100, 10 % glycerol, 0.5 mM PMSF and protease inhibitor. Protein concentration was measured by the Bradford assay. Forty micrograms of total protein were separated by 8, 12.5 or 15 % SDS-PAGE and then transferred to a nitrocellulose membrane, which was blocked with TBS-T/5 % BSA. Incubation with primary antibodies of interest was performed overnight, at 4 °C. Rabbit Naa40 antibody was kindly provided by Dr. Qiwei Zhai [49]. The rabbit polyclonal N-acH4 antibody was raised against the acNH-SGRGKGGKGLGKC antigen using the Eurogentec ‘Speedy 28-day polyclonal antibody’ service (Belgium). The modification-specific antibody was isolated by affinity purification of total serum through the specific N-terminally acetylated H4 peptide. To eliminate immunoglobulins that recognise the backbone of H4, the bound fraction from the above purification was further cleaned up by passing it through a column consisting of the corresponding ‘unmodified’ version of the peptide. The p53 (DO-1), Bcl-2 and β-actin antibodies were purchased from Santa Cruz Biotechnology Inc. PARP-1, Bax, caspase-3, -6, -7, -8, and -9 antibodies were purchased from Cell Signalling Technology (Danvers, Massachusetts, USA). H4 antibody was purchased from Millipore and GAPDH and fibronectin antibodies were purchased from Abcam. Histones purified from calf thymus were purchased from Sigma. Immunoreactive bands were visualized with the enhanced chemiluminescence system. The intensity values from the densitometry analysis were normalised against GAPDH or β-actin using Image J analysis software (NIH). Intensity values were expressed as fold change compared to control.

### MTT proliferation assay

Cells were seeded at a concentration of 5 × 10^3^ cells per well (HCT116 and HT-29) or 1 × 10^4^ cells per well (STO cells) in a 96-well plate and incubated with 7.5 nM of siRNAs (HCT116 and STO cells) or 25 nM of siRNAs (HT-29 cell), for different time periods (24, 48 or 72 h). MTT dye (1 mg/ml) was added to each well at the end of the incubation period and cells were incubated for 4 h at 37 °C. Formazan product was dissolved in DMSO and the plate was incubated on a plate shaker at 250 rpm for 30 min. The absorbance was measured at 570 nm using Perkin Elmer Wallac Victor 1420-002 Multilabel Counter and was proportional to the number of viable cells per well.

### Cell cycle analysis

Cells were seeded in 6-well plates or in 100 mm dishes so that they will be ~30 % confluent by the time of transfection. After siRNA treatment for 24, 48 or 72 h, cells were harvested, washed in 1× PBS and fixed in ice-cold 70 % ethanol. Cells were left at 4 °C overnight and the next day were pelleted, washed in 1× PBS and resuspended in PI staining solution (containing 0.02 mg/ml Propidium Iodide and 0.2 mg RNase A). After a 45 min incubation at 37 °C, the samples were transferred to FACS tubes and analysed with the “Guava EasyCyteTM” flow cytometer and the “GuavaSoft” analysis software (Millipore, Watford, UK).

### Annexin V/PI staining assay

Annexin V/PI staining was performed using Tali Apoptosis Kit (Invitrogen A10788). After treatment, cells were trypsinised, centrifuged for 15 min at 1000 rpm (95rcf) and cell pellet was dissolved in 100 µl Annexin binding buffer (5 × 10^5^–5 × 10^6^ cells/ml). To each 100 µl of samples, 5 µl of annexin V were added and after thorough mixing, samples were incubated at RT for 20 min. Cells were then centrifuged for 15 min at 1000 rpm (95 rcf) and supernatant was discarded. Cell pellet was resuspended in 100 µl of Annexin binding buffer and 1 µl of Propidium Iodide (PI). After incubating the samples for 5 min at RT in the dark, they were transferred in Tali cellular analysis slides. Analysis of apoptosis was performed using Tali image-based cytometer (Invitrogen). The annexin V positive/PI negative cells were recognized as early apoptotic cells by the cytometer software whereas the annexin V positive/PI positive cells were identified as late apoptotic/dead cells. Similarly, the annexin V negative/PI negative cells were identified as viable cells. The baseline apoptosis varied between 5 and 15 % among the various apoptosis-related experiments performed.

### Phase contrast microscopy

Images were acquired using ZeissAxio Observer.A1 microscope and ZeissAxioVision 4.8.2 software.

### Immunofluorescence

Cells were grown on poly-l-lysine coated coverslips, in a 6-well plate. At the end of the incubation, cells were washed 2 times with 1× PBS and then fixed at RT, for 10 min in 4 % paraformaldehyde solution in 1× PBS. Cells were washed 2 × 5 min with 1× PBS and then permeabilized using 0.5 % Triton-X solution in PBS for 10 min. After 2 × 5 min washes with 1× PBS permeabilised cells were blocked for 45 min in PBG (0.2 % cold water fish gelatin, 0.5 % BSA in 1× PBS) and then incubated with primary antibody diluted in PBG, for 2 h, at RT, in the dark. Cells were then washed for 3 × 5 min with PBG. Incubation with secondary antibody diluted in PBG followed, for 45 min at RT, in the dark. After 2 × 5 min washes with PBG, cover slips were placed to microscope slides with mounting media (prolong Gold). Nuclei were stained with Hoechst dye (33342, Invitrogen). Anti-Rabbit FITC antibody was purchased from Jackson ImmunoResearch. All images were acquired using a ZeissAxio Observer.A1 microscope.

### Statistical analysis

Data represent measurements of three independent experiments and values are expressed as mean ± SD. Statistical analysis was carried out using “Mstat” software [version 5.5.3, McArdle Laboratory for Cancer Research, University of Wisconsin—Madison (http://mcardle.oncology.wisc.edu/mstat/)]. Results are compared using a Wilcoxon rank-sum test. For all statistical tests differences are considered significant at *p* ≤ 0.05 (*).

## Results

### Naa40 knockdown in HCT116 cancer cells reduces ribosomal RNA expression and cell viability

**Fig. 1:**
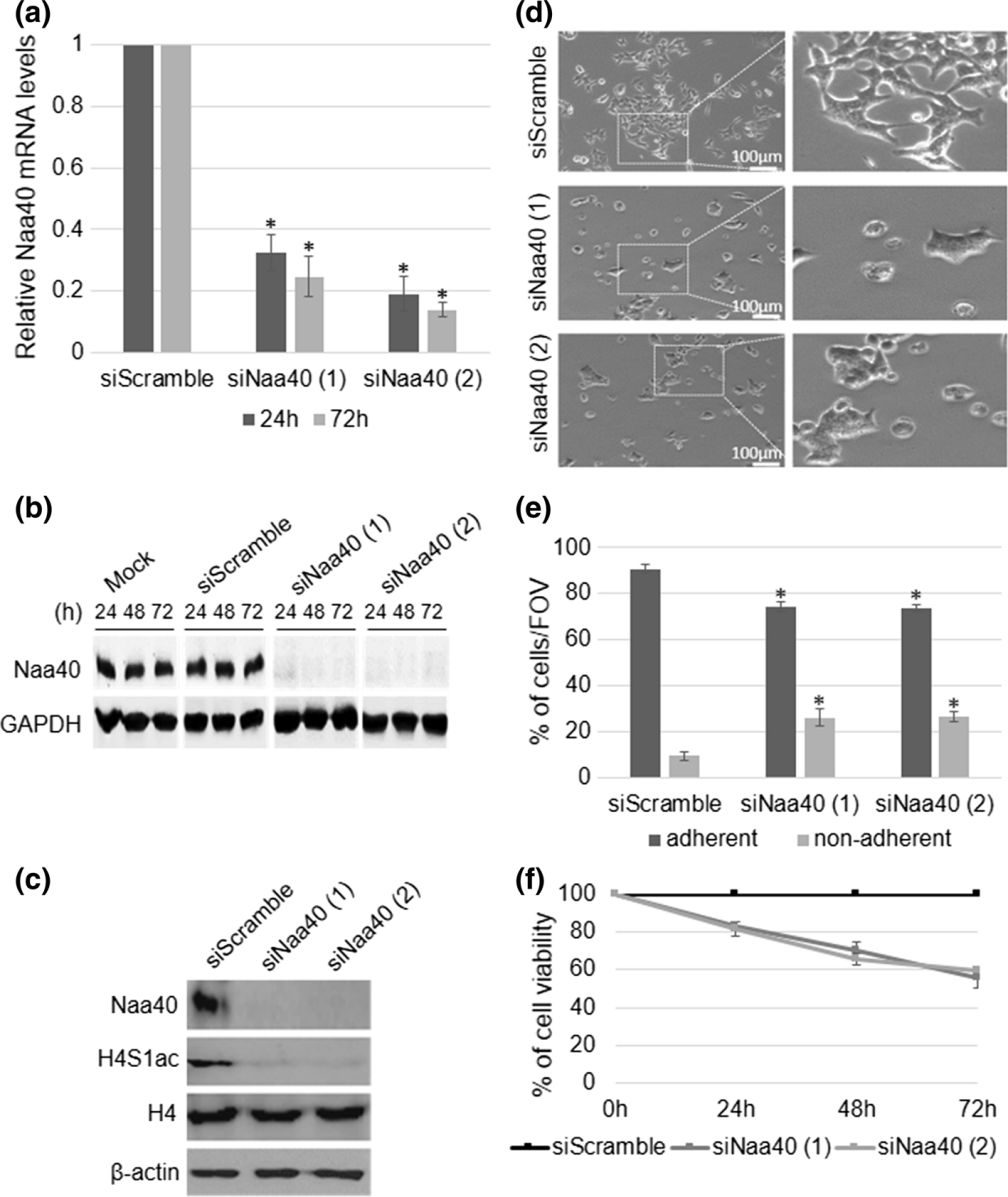
Naa40 knockdown reduces cell survival in HCT116 cells. Colorectal cancer HCT116 cells were transfected with 7.5 nm of Naa40 siRNA-1, Naa40 siRNA-2 or scramble siRNA and incubated for 24–72 h. **a** RT-PCR analysis of whole cell lysates. The levels of Naa40 mRNA were quantified and then normalised to the levels of β-actin mRNA, whose expression remained unchanged. **b** Immunoblot analysis to determine Naa40 protein levels in whole cell extracts. Mock represents sample in which cells were subjected to the transfection procedure in the absence of any siRNA. GAPDH levels were monitored and used as a loading control. **c** Immunoblot analysis using antibodies against Naa40, N-terminal acetylation of histone H4 (N-acH4), total histone H4 and β-actin (72 h). **d** Phase contrast microscopy analysis of scrambled siRNA and Naa40 siRNA treated cells (48 h) (*scale bar* 100 µm). *Dashed rectangles* represent the zoomed-in regions that are shown on the *right panels*. The images are representative fields of view from at least three independent experiments. **e** Quantitation of the percent of adherent and non-adherent cells per field of view, in siScramble and siNaa40 conditions (48 h). Data represent the mean number of 15 fields of view taken from three independent experiments ±SD (*p* value <0.05) **f** MTT cell viability assay. Cell viability is shown as a percentage relative to the scramble-siRNA control. The data represent the mean of three replicates and are representative of at least three different experiments ±SD (*p* value <0.02). Western blot images in **b**, **c** are representative of at least three experiments

In a previous study we have shown that loss of yeast Naa40 reduces transcription at the ribosomal DNA locus [33]. To investigate whether human Naa40 has the same effect on ribosomal RNA (rRNA) expression, we depleted Naa40 in HCT116 colorectal cancer cells using two different siRNAs. Both siRNAs reduced the mRNA and protein levels of Naa40 significantly and to the same extent. The depletion was already robust at 24 h and was sustained until 72 h after Naa40-siRNA transfection (Fig. 1a, b). A negative siRNA control (scramble siRNA) did not alter the levels of Naa40 compared to mock-treated sample (Fig. 1b), indicating that the achieved knockdown using the targeting Naa40 siRNAs was specific. To confirm the above knockdown, we also examined the levels of endogenous N-terminal acetylation of histone H4 (N-acH4), which is specifically catalysed by Naa40, using an antibody that we have previously developed [33]. The N-acH4 antibody recognises specifically histone H4 when is acetylated at its N-terminus, as indicated by western and dot-blot analysis (Supp data 2). In agreement with the above knockdown results, the levels of N-acH4 were significantly decreased in cells treated with both Naa40-siRNAs (Fig. 1c). As expected, the levels of N-acH4 did not change in the scramble-siRNA treated cells (Fig. 1c). Naa40 depletion did not affect the levels of total H4 (Fig. 1c) demonstrating that only the N-terminally acetylated fraction is decreased. These findings indicate that RNAi achieved an efficient and sustained depletion of Naa40 in HCT116 cells that also leads to reduction of its substrate N-acH4.

We then proceeded to determine whether depletion of Naa40 in HCT116 cells affects rRNA expression. To do this, we examined the levels of different ribosomal RNAs (28S, 18S, 5.8S as well as their precursor 45S) by real time-PCR, at 24 and 72 h post-transfection. The expression of rRNA transcripts was significantly decreased upon Naa40 knockdown and this reduction was consistent in both Naa40-siRNA treatments (Supp data 3). These results suggest that human Naa40, similarly to its yeast homolog [33], has a role in regulating rDNA expression.

While performing the above knockdown experiments, we observed extensive loss of viable cells in the Naa40-siRNA transfected cell population compared to the scramble-siRNA control. This decreased viability was maintained throughout the 72 h time course of the siRNA treatment. Specifically, the cells treated with the Naa40 siRNAs showed decreased colony formation and morphological characteristics of dead cells, including cellular rounding and detachment (Fig. 1d; Supp data 4). To quantify these observations we first counted adherent and non-adherent cells, representing the viable and dead cells respectively, in several microscopic fields of view. We found at least a twofold increase in the number of non-adherent cells upon depletion of Naa40 (Fig. 1e), indicative of enhanced cell death. To validate these results MTT assay was also performed to quantify cell viability more accurately. Cells that were depleted in Naa40 showed a significant reduction in survival compared to control cells with the highest decrease (40 %) in cell viability occurring at 72 h post-transfection (Fig. 1e). Overall, these results indicate that Naa40 is required for HCT116 cell survival.

### Depletion of Naa40 triggers apoptosis

**Fig. 2:**
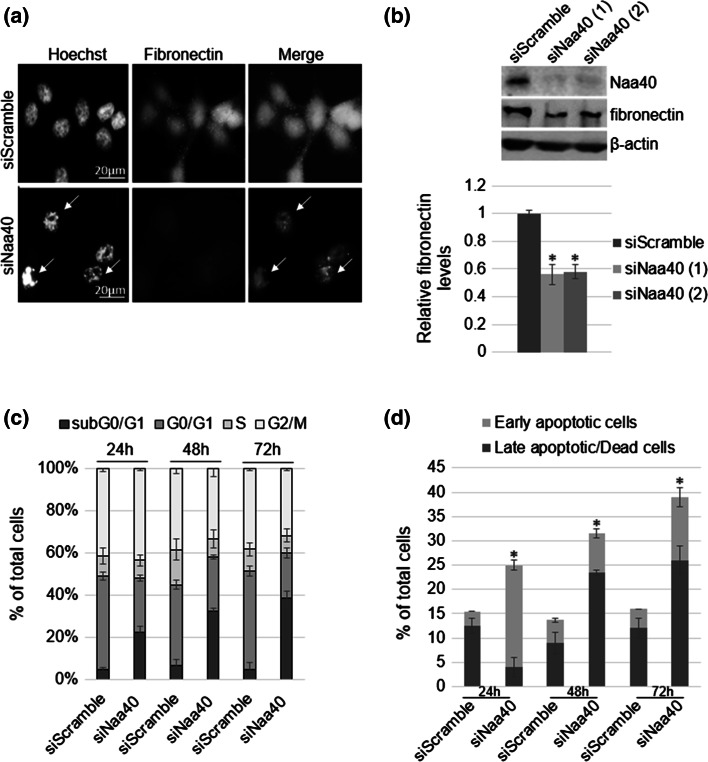
Depletion of Naa40 induces apoptosis in HCT116 cells. **a** Representative immunofluorescence pictures of HCT116 cells transfected with Naa40-siRNA or a scramble-siRNA for 72 h. Green-FITC staining was used against fibronectin and blue Hoechst staining was used to visualise the nuclei. *Arrows* indicate apoptotic cells (*scale bar* 20 µm). **b** Immunoblot analysis of fibronectin levels in the absence of Naa40 (72 h post-transfection). β-actin was used as a control. Densitometry analysis (*bottom panel*) shows the levels of fibronectin in siNaa40 treatments relative to the scramble-siRNA sample. **c** Cell-cycle analysis of HCT116 cells after treatment with a scramble-siRNA or Naa40-siRNA for 24, 48 and 72 h. **d** Annexin V/PI staining was used to assess the apoptotic cell population. Images in **a**, **b** are representative of three independent experiments. Statistically significant changes (*p* value <0.02) in **b**, **d** are indicated by an *asterisk* (*). The data in **b**–**d** represent the mean of at least three replicates and are representative of at least three different experiments ±SD (*p* value <0.02)

The significant decrease of viable cells (Fig. 1e, f) raised the hypothesis that depletion of Naa40 induces cell death. Additionally, this hypothesis is supported by the fact that the morphological changes observed in Fig. 1d comprise a characteristic of apoptotic cells, which often occur due to loss of cytoskeletal and cell-adhesion proteins. Fibronectin is one such adhesion molecule whose reduction has been linked to loss of cell survival and induction of apoptosis [50, 51]. Our data indicate that knockdown of Naa40 reduced fibronectin protein levels, as shown by immunofluorescence and western-blot analyses (Fig. 2a, b). Consistent with the loss of fibronectin and the observed cellular morphological changes, Hoechst staining shows that siNaa40-treated cells are more brightly stained compared to control cells indicating nuclear condensation which is another key feature of apoptotic cells (Fig. 2a).

To further support our suspicion that Naa40 knockdown induces apoptosis, we then analysed cells by flow cytometry at 24, 48 and 72 h after Naa40 knockdown. Starting at the 24 h timepoint we observed a significant increase of the subG0/G1 fraction in the Naa40 treated cells (22.3 %) compared to the corresponding fraction (4.7 %) in the control treatment (Fig. 2c). The fraction of cells in the subG0/G1 phase, which represents the apoptotic population, was progressively increased upon siNaa40 transfection compared to the siScramble control, reaching up to 38.7 % of the total cell population (Fig. 2c). Finally, to corroborate the results of the cell-cycle analysis we have also examined the induction of apoptosis by analysing siRNA transfected cells with dual staining for Annexin V and propidium iodide (PI). At 24 h post siNaa40 transfection, a significant accumulation of early apoptotic cells was detected, while at 48 and 72 h post-transfection a progressive increase in the late apoptotic/dead cell population was observed (Fig. 2d). These results demonstrate that Naa40 depletion mediates cell death through apoptosis and not necrosis. The percentage of early versus late apoptotic cells remained approximately the same in the control sample throughout the time course of the treatment, confirming that apoptosis is specifically induced upon Naa40 knockdown (Fig. 2d). Taken together, our data show that loss of Naa40 induces apoptosis in HCT116 colorectal cancer cells.

To determine whether induction of apoptosis upon Naa40-knockdown is specific to HCT116 cells, we also depleted this enzyme in another colorectal cancer cell line (HT-29) and non-cancerous mouse embryonic (STO) fibroblasts (Supp data 5a). Similarly to HCT116, depletion of Naa40 in HT-29 cells using two distinct siRNAs reduced cell viability approximately by 40 % at 72 h post-transfection (Supp data 5b). In contrast, when Naa40 was depleted in STO mouse embryonic fibroblasts (Supp data 5a), no significant change on the percentage of viable cells was observed at 72 h post-transfection compared to the control treatment (Supp data 5b). Furthermore, downregulation of Naa40 in HT-29 cells resulted in an almost threefold increase of the subG0/G1 fraction when these cells were treated with the two separate siNaa40-RNAs (34.4 and 30.9 % respectively) as compared to the siScramble (11.5 %) control (Supp data 5c). Altogether, these results suggest that Naa40 depletion induces apoptosis in colorectal cancer cells, but does not affect the viability of non-malignant cells.

### Naa40 knockdown-mediated apoptosis is caspase-dependent

**Fig. 3:**
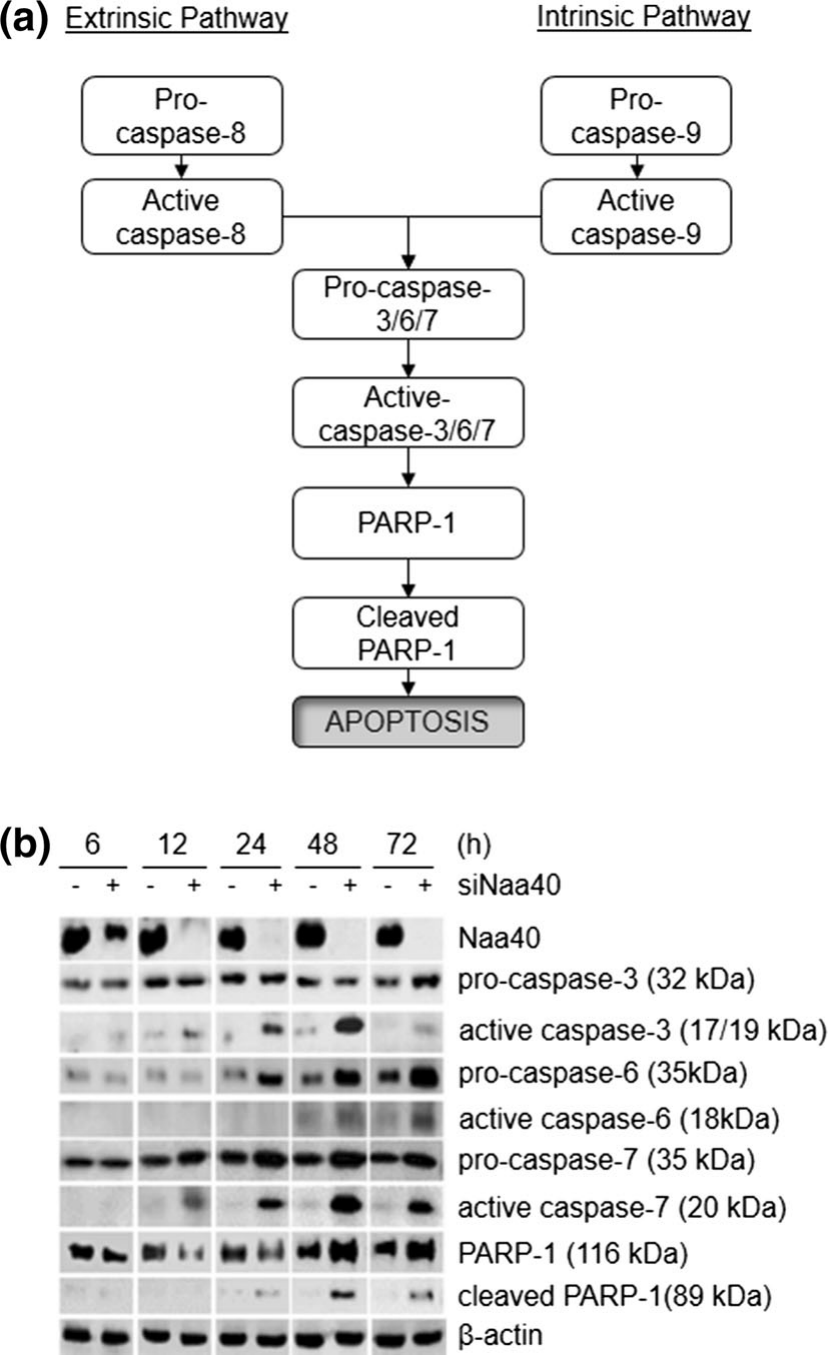
Depletion of Naa40 stimulates apoptosis through a caspase-dependent mechanism. **a** Schematic illustrating caspase-dependent apoptotic pathways. The signalling cascade could be induced by either the extrinsic or intrinsic apoptotic pathway through cleavage of the initiator caspase-8 or casapse-9, respectively. In turn, these caspases activate common executioner caspases (caspases-3/6/7) that subsequently cleave PARP-1 and lead to apoptosis. **b** Immunoblot analysis of scramble-siRNA (−) or Naa40-siRNA (+) transfected HCT116 cells during a timecourse of 6–72 h. Antibodies were used against Naa40, caspase-3, -6, -7, PARP-1 and β-actin, which served as a loading control. The image is representative of at least three independent experiments

To determine the apoptotic pathway that is induced by depletion of Naa40 in colon cancer cells, we first investigated its effect on caspase activation. We focused on effector caspases 3, 6 and 7 as well as their substrate PARP-1 which are all cleaved and activated upon signalling from either the intrinsic or extrinsic apoptotic pathways (Fig. 3a) [52]. Hence, the levels of cleaved executioner caspases and PARP-1 were examined in cells transfected with siRNAs during a 6–72 h timecourse. We found that when Naa40 was maximally reduced at 12 h post-transfection the active forms of caspases-3 and -7 began to accumulate. Moreover, the apoptotic PARP-1 fragment (89 kDa) was detected soon after the cleavage of the executioner caspases (Fig. 3b). Cleavage of caspase-6 was also observed at 48 h post treatment with siNaa40. Cells transfected with the siScramble control did not show significant accumulation for any of the cleaved forms of caspases or PARP-1 suggesting that their activation is specific to Naa40 depletion (Fig. 3b).

In addition to the active caspases, an upregulation of the pro-caspases was also observed when Naa40 was depleted (Fig. 3b). Overexpression of pro-caspases has been previously reported to occur during stimulation of apoptosis. It was suggested that pro-caspase upregulation is necessary in order to have sufficient amounts of these precursors within the cells which are needed to further stimulate and complete the apoptotic process [53–55]. We have also observed an upregulation of full-length PARP-1 at the later timepoints (48 and 72 h) of the siNaa40 treatment (Fig. 3b). Expression of full-length PARP-1 has been linked with induction of cell death as a response to excessive DNA damage [56, 57], which is probably taking place within HCT116 apoptotic cells after 48 and 72 h of siNaa40 treatment. The increased levels in pro-caspases and PARP-1 raised the possibility that depletion of Naa40 and perhaps its associated N-acH4 modification might regulate their expression at the transcriptional level. To test this possibility, we performed RT-PCR analysis to quantify the mRNA levels of all pro-caspases and PARP-1. The mRNA levels of all these proteins, with the exception of pro-capsase-7, did not change in the absence of Naa40 when compared to the siScramble control treatment (Supp data 6). Therefore, depletion of Naa40 and thus, loss of histone N-terminal acetylation do not directly affect the promoter of these genes and point towards a post-transcriptional rather than a transcriptional regulation. Collectively, our results verify the conclusion that Naa40 depletion triggers apoptosis in colon cancer cells and reveal the involvement of caspases within this process.

### Induction of apoptosis in Naa40-depleted cells requires activation of the mitochondrial/intrinsic pathway

**Fig. 4:**
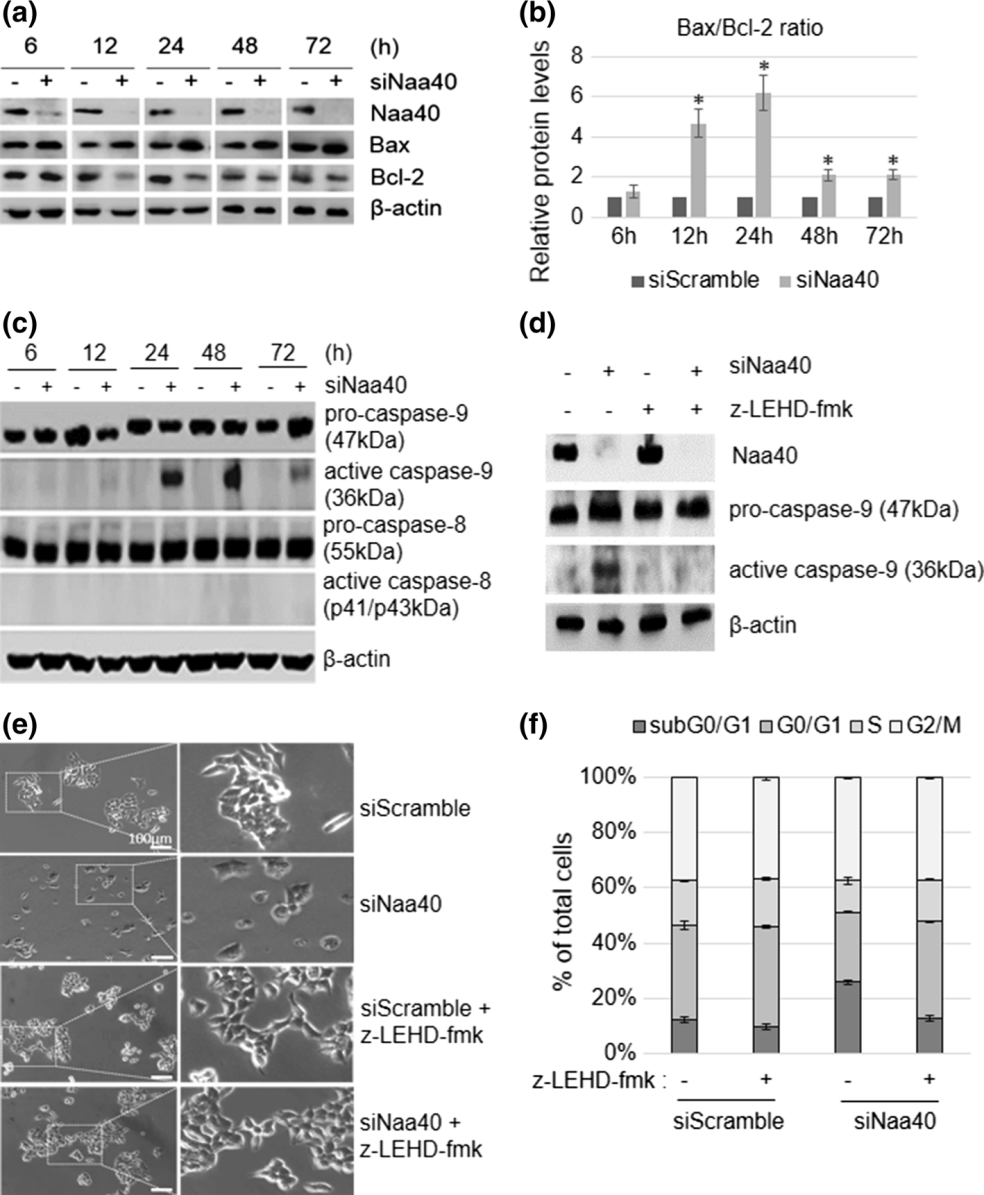
Naa40-knockdown induces the mitochondrial apoptotic pathway through activation of caspase-9. **a** Immunoblot analysis of untransfected (−) or Naa40-siRNA (+) transfected HCT116 cells during a timecourse of 6–72 h using antibodies against Naa40, Bax, Bcl-2 and β-actin, as a loading control. **b** Quantitative densitometry showing the ratio between Bax and Bcl-2 protein levels according to western blots in (**a**). An *asterisk* indicates statistically significant changes (*p* value <0.02). **c** Immunoblot analysis as in (**a**) using antibodies against caspase-9, -8 and β-actin. **d** Immunoblot analysis of HCT116 cells transfected with siScramble (−) or siNaa40 (+) in the presence (+) or absence (−) of caspase-9 inhibitor z-LEHD-fmk. Whole cell lysates were collected 48 h post-transfection and assayed using antibodies against caspase-9 and β-actin. **e** Representative phase contrast images from at least three independent experiments showing HCT116 cells treated as in (**d**) for 48 h (*scale bar* 100 µm). Dashed rectangles represent the zoomed-in regions that are shown on the *right panels*. **f** Cell cycle analysis of cells treated as in (**d**) for 48 h. Western blot images in **a**, **c**, **d** are representative of three independent reproducible experiments. Values in **b**, **f** represent the mean of three independent experiments ±SD (*p* value <0.02)

Since the effector caspases and PARP-1 are found downstream of both the intrinsic and extrinsic apoptotic pathways (Fig. 3a), we next sought to determine which pathway is specifically activated when Naa40 is depleted. To start exploring this, we first examined the levels of Bax and Bcl-2 in siRNA transfected cells. Upon Naa40 depletion we detected an increase in the levels of the pro-apoptotic factor Bax and a decrease in the levels of the anti-apoptotic protein Bcl-2 (Fig. 4a). Additionally, the ratio between Bax/Bcl-2 levels was gradually increasing until 24 h after siNaa40 transfection and remained more than double for 72 h (Fig. 4b), implicating the mitochondrial pathway in the induction of apoptosis.

To determine whether the mitochondrial pathway is the only pathway activated upon Naa40 downregulation, we next examined the cleavage of the initiator caspases-8 and -9 in HCT116 cells, which, respectively, stimulate specifically the extrinsic and intrinsic apoptotic pathways (Fig. 3a). We detected cleavage of caspase-9 when Naa40 was depleted from the cells (Fig. 4c) and this coincided with the cleavage of the effector caspases (compare Figs. 3b, 4c). Caspase-9 activation was also observed in HT-29 cells after Naa40 depletion (Supp data 5d). These results suggest that the mitochondrial pathway could be the main route through which Naa40 knockdown promotes programmed cell death.

To conclusively demonstrate the requirement of the mitochondrial pathway for the induction of apoptosis by Naa40 knockdown, we monitored apoptosis of Naa40-depleted HCT116 cells in the presence of the irreversible caspase-9 inhibitor, z-LEHD-fmk. Incubation of HCT116 cells with z-LEHD-fmk prevented the cleavage of caspase-9 after depletion of Naa40 (Fig. 4d), as expected. Notably, cells depleted of Naa40 in the presence of the caspase-9 inhibitor showed similar morphology and growth as the siScramble transfected cells, suggesting that apoptosis induced by Naa40 knockdown was prevented (Fig. 4e; Supp data 7a). Quantification of cells from different microscopic fields of view also showed that Naa40 depletion in the presence of the capsase-9 inhibitor does not increase the number of non-adherent cells (7.2 %) relative to the siScramble control (8 %), but a significant increase is observed, as expected, in the siNaa40 only treatment (21 %) (Supp data 7b). The lack of siNaa40-induced apoptosis in the presence of the capsase-9 inhibitor was further verified by cell cycle analysis. Treatment with the caspase-9 inhibitor reduced significantly the apoptotic subG0/G1 population (from 25.95 to 12.8 %) and restored the G1-phase fraction in the Naa40 depleted cells (from 25 to 35 %) (Fig. 4f). Taken together, these results demonstrate that activation of caspase-9 and thus, the mitochondrial pathway are essential for the induction of apoptosis in colon cancer cells depleted of Naa40.

### Depletion of Naa40 promotes apoptosis in a p53-independent manner

**Fig. 5:**
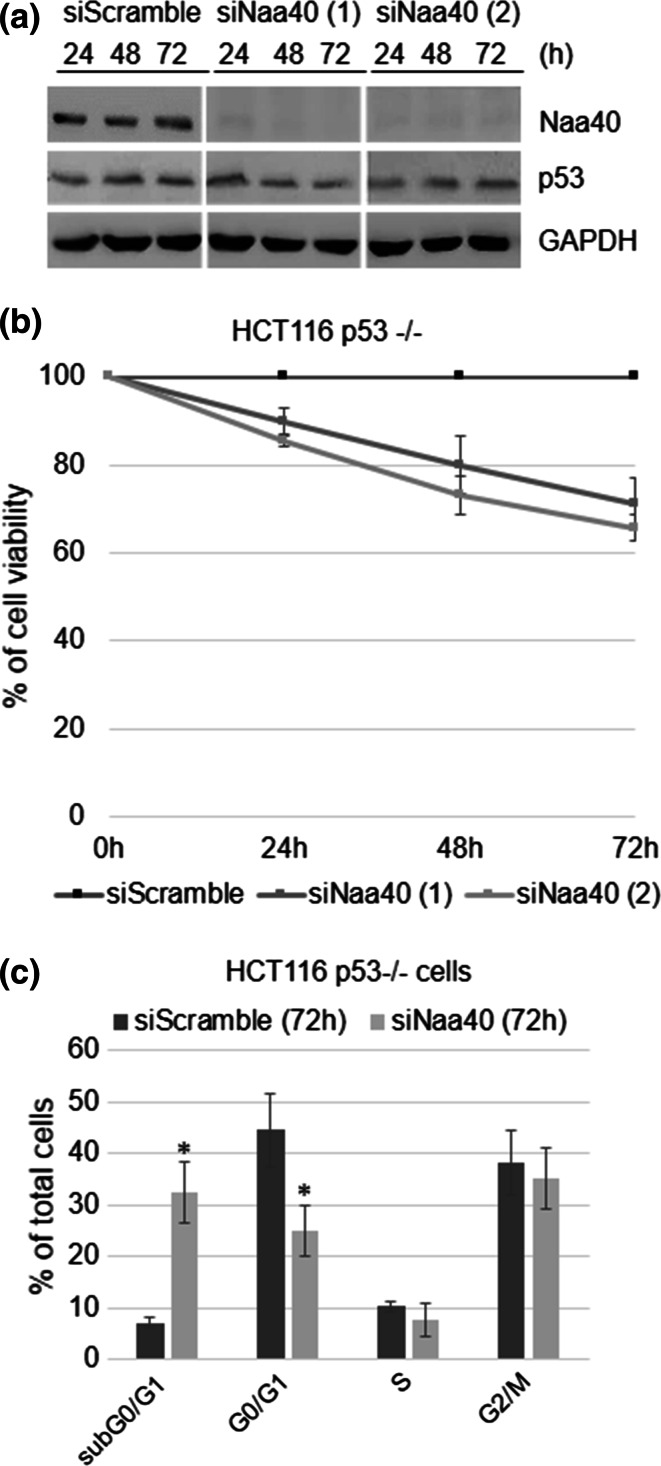
Naa40-knockdown induces apoptosis in HCT116 cells independently of p53 status. **a** Immunoblot analysis of whole cell lysates prepared from HCT116 cells transfected with scramble-siRNA or Naa40-siRNAs for 24, 48 and 72 h using antibodies against p53, Naa40 and GAPDH, as a loading control. Representative western blot is shown from three independent experiments. **b** MTT cell viability assay was performed in p53 null cells (HCT116 p53−/−) transfected with scramble-siRNA or Naa40-siRNAs for 24, 48 and 72 h. Cell viability is shown as a percentage relative to the siScramble control. **c** Cell-cycle analysis of HCT116 p53−/− cells treated with a scramble-siRNA or Naa40-siRNA for 72 h. *Error bars* indicate standard deviation of triplicate experiments ±SD (*p* value <0.02). Statistically significant changes (*p* value <0.02) are indicated by an *asterisk* (*)

One key regulator of apoptosis is the p53 tumour suppressor, which is frequently inactivated in colon cancer cells [58]. Therefore, we wanted to ascertain whether this tumour suppressor plays a role in the induction of apoptosis once Naa40 is depleted. To do this, we initially compared the p53 protein levels between cells transfected with siNaa40 and the siScramble control. We found that Naa40 depletion in HCT116 cells had no effect on p53 protein levels, as the signal was the same between the siNaa40 and siScramble treatments (Fig. 5a). This result raised the possibility that p53 is not involved in the apoptosis induced by Naa40 knockdown.

To examine this possibility, we depleted Naa40 in HCT116 cells that lack p53 protein (HCT116 p53−/−) (Supp data 8) and examined cell viability and apoptosis. Lack of p53 in HCT116 cells did not impede the effects of Naa40 depletion. After 72 h of siNaa40 treatment using two distinct siRNAs there was about a 40 % reduction of viable cells (Fig. 5b) and approximately 35 % increase of the subG0/G1 cellular fraction, representing the apoptotic population (Fig. 5c). Notably, the percentage of cells that undergo apoptosis in HCT116 p53−/− cells upon Naa40 depletion is similar to the apoptotic fraction observed in p53+/+ HCT116 cells (compare Fig. 1f with 5b and Fig. 2c with 5c). Consistently, we observed a significant induction of siNaa40-mediated apoptosis in HT-29 colon cancer cells (Supp data 5) in which canonical p53 function is defective due to the presence of an oncogenic missense mutation within its DNA-binding domain [59]. Altogether, our data show that p53 is not required for the apoptosis induced by Naa40 knockdown.

## Discussion

Altering the levels of NATs in cancer cells results in cell-cycle arrest or cell-death indicating that these enzymes are important candidate targets for cancer therapy [18]. Here we focused on one of the most substrate-selective NAT enzymes, Naa40, which so far is known to acetylate only the N-termini of histones H4 and H2A [28]. We previously revealed a role of yeast Naa40 in the control of ribosomal RNA transcription. More specifically, deletion of the acetyltransferase resulted in decreased ribosomal RNA expression, as shown by real time-PCR analysis [33]. This is in line with our current data which show that upon human Naa40 depletion, the amount of all rRNAs is significantly reduced (Supp data 3). Our results suggest that Naa40 probably utilises a conserved mechanism to control gene activation in eukaryotes. Mainly, in the present study, we uncover a new link between hNaa40 and colorectal cancers. We show that depletion of Naa40 in colon cancer cells triggers a strong apoptotic response. The significant increase in the subG0/G1 cell population (Fig. 2; Supp data 5) together with the transition of cells from early to late apoptosis show clearly that Naa40-knockdown induces apoptosis and not necrosis. The induction of apoptosis upon Naa40 depletion is conclusively demonstrated by the fact that executioner caspases are activated (Fig. 3b) and inhibition of the initiating caspase-9 impedes the apoptotic effect of Naa40-knockdown (Fig. 4d–f). The latter results also demonstrate that the programmed cell-death observed in Naa40-knockdown cells is exclusively signalled by the mitochondrial, or intrinsic, apoptotic pathway. Moreover, the link of Naa40 knockdown to apoptosis appears to be specific to cancer cells since depletion of Naa40 in non-malignant embryonic fibroblasts does not stimulate cell death (Supp data 5). Overall, these data associate Naa40 to colorectal cancer development and highlight the potential exploitation of Naa40 as a therapeutic target.

This study suggests that the activity of Naa40 maintains the balance between cell survival and cell-death. The fact that Naa40 acetyltransferase activity is specifically targeted towards histone proteins and possibly towards SMARCD2 [30], which is another chromatin related protein, postulates that Naa40 influences cell survival by regulating gene expression and not through direct protein N-terminal acetylation of key regulators of cell growth and/or apoptosis. The diminished ribosomal RNA transcription observed upon Naa40 depletion, in both human and yeast cells, enhances this notion ([33]; Supp data 3). Our results imply that ribosomal DNA silencing and consequently impaired ribosome biogenesis as a result of Naa40 loss, could be the triggers for inducing apoptosis in HCT116 and HT-29 colon cancer cells. Consistent with such a scenario, inhibition of PolI that is required for rRNA synthesis promotes programmed cell-death in cancer cells derived from solid tumours through a p53-independent process [60].

The present data reveal an anti-apoptotic role for Naa40 in colorectal cancer cells, highlighting the importance of this enzyme in human cells. Naa40 was previously linked to apoptosis but, in contrast to our findings, it was shown that siRNA mediated knockdown of Naa40 protects hepatoma cells from drug-induced apoptosis [49]. A similar phenomenon has also been reported for another NAT, namely Naa10, which has been ascribed roles both as an anti- and pro-apoptotic factor. For example, knockdown of Naa10 in HCT116 and HeLa cells caused caspase-dependent apoptosis [61, 62], while depletion of Naa10 in other cell lines, like U2OS and HT1080, conferred resistance to DNA-damage induced apoptosis. It has been proposed that these opposing roles are due to the fact that Naa10 has numerous protein substrates [63] and employs different mechanisms to exert its function, which can vary among cell-types and pathological contexts [18, 27]. An analogous rational could perhaps explain the contrasting roles of Naa40 in apoptosis. With more than 90 % of histone H4 proteins N-terminally acetylated within a cell [64], it is likely that Naa40 impacts chromatin structure and transcription at various genomic loci. Therefore, knockdown of Naa40 might affect distinct sets of genes in different cell types and this could explain the opposing effects of Naa40 on cell survival. Future studies in various biological contexts will provide new insights for resolving the exact apoptotic role of Naa40.

Similarly to other NATs [18], Naa40 has emerged through this study as a potential therapeutic target because its inhibition could induce apoptosis in cancer cells and hence hinder carcinogenesis. Therefore, NAT specific inhibitors could be valuable epigenetic anticancer therapeutics. Specifically, a first generation of NAT compounds has been developed that can selectively target the NatA complex, its catalytic subunit Naa10 or the enzyme NatE/Naa50 [65]. Epigenetic therapies for cancer treatment hold great promise and future efforts may add Naa40 to the repertoire of epigenetic regulators that are currently being targeted by small-molecule inhibitors in preclinical and clinical studies. The recently defined structure of Naa40 showed that although the overall fold is similar to other NATs, it also contains unique structural features, such as in the loops forming the substrate-binding site [30], which can be exploited to develop specific Naa40 inhibitors. The findings within the current study support the inhibition of Naa40 as a viable therapy because of three main reasons. Firstly, Naa40 knockdown in colon cancer cells induces a strong apoptotic effect but depletion in noncancerous mouse embryonic fibroblasts does not affect cell viability (Fig. 2; Supp data 5). Secondly, the siNaa40-mediated induction of apoptosis in HCT116 cancer cells is irrespective of the p53 status (Fig. 5). Since the gene encoding p53 is found mutated in 40–50 % of colorectal cancers [58], a comprehensive therapeutic regimen should avoid the requirement of having intact endogenous p53 function. Thirdly, knockdown of Naa40 alters the levels of Bcl-2 family members and its effect is completely driven through the mitochondrial apoptotic pathway since the caspase-9 inhibitor (z-LEHD-fmk) blocked apoptosis in siNaa40-treated cells (Fig. 4). Notably, the mitochondrial apoptotic pathway has a prominent role in chemotherapy effectiveness and therefore, it is considered a favourable target for cancer therapy [66].

In summary, our findings reveal a conserved role of hNaa40 in the control of rRNA expression and, mainly, link Naa40 to cancer cell apoptosis. Knockdown of Naa40 in HCT116 and HT-29 colon cancer cells affects their survival by caspase-9 activation. In addition, execution of apoptosis in HCT116 cells is shown to be p53-independent. Taking into account that loss of Naa40 has no effect on cell viability of noncancerous embryonic fibroblasts, the present study identifies Naa40 inhibition as a potential epigenetic therapeutic strategy, which could be used in the treatment of colon cancers carrying either wild-type or mutant p53.

## Electronic supplementary material

Supplementary material 1 (DOCX 2223 kb)

## Abbreviations

NAT: N-terminal acetyltransferase
Naa40: N(Alpha)-Acetyltransferase 40
N-acH4: N-terminal acetylation of histone H4
MOMP: Mitochondrial membrane permeabilisation
PI: Propidium iodide
MTT: 3-(4,5-Dimethyl-2-yl)-2,5-diphenyltetrazolium
rRNA: Ribosomal RNA

## References

[1] Kouzarides T (2007). Chromatin modifications and their function. Cell.

[2] Seo J, Lee KJ (2004). Post-translational modifications and their biological functions: proteomic analysis and systematic approaches. J Biochem Mol Biol.

[3] Arnesen T, Van Damme P, Polevoda B, Helsens K, Evjenth R, Colaert N (2009). Proteomics analyses reveal the evolutionary conservation and divergence of N-terminal acetyltransferases from yeast and humans. Proc Natl Acad Sci USA.

[4] Brown JL, Roberts WK (1976). Evidence that approximately eighty per cent of the soluble proteins from Ehrlich ascites cells are Nalpha-acetylated. J Biol Chem.

[5] Hershko A, Heller H, Eytan E, Kaklij G, Rose IA (1984). Role of the alpha-amino group of protein in ubiquitin-mediated protein breakdown. Proc Natl Acad Sci USA.

[6] Jornvall H (1975). Acetylation of protein N-terminal amino groups structural observations on alpha-amino acetylated proteins. J Theor Biol.

[7] Hwang CS, Shemorry A, Varshavsky A (2010). N-terminal acetylation of cellular proteins creates specific degradation signals. Science.

[8] Park SE, Kim JM, Seok OH, Cho H, Wadas B, Kim SY (2015). Control of mammalian G protein signaling by N-terminal acetylation and the N-end rule pathway. Science.

[9] Arnesen T (2011). Towards a functional understanding of protein N-terminal acetylation. PLoS Biol.

[10] Forte GM, Pool MR, Stirling CJ (2011). N-terminal acetylation inhibits protein targeting to the endoplasmic reticulum. PLoS Biol.

[11] Hollebeke J, Van Damme P, Gevaert K (2012). N-terminal acetylation and other functions of Nalpha-acetyltransferases. Biol Chem.

[12] Silva RD, Martinho RG (2015). Developmental roles of protein N-terminal acetylation. Proteomics.

[13] Tooley JG, Schaner Tooley CE (2014). New roles for old modifications: emerging roles of N-terminal post-translational modifications in development and disease. Protein Sci.

[14] Ree R, Myklebust LM, Thiel P, Foyn H, Fladmark KE, Arnesen T (2015). The N-terminal acetyltransferase Naa10 is essential for zebrafish development. Biosci Rep.

[15] Polevoda B, Brown S, Cardillo TS, Rigby S, Sherman F (2008). Yeast N(alpha)-terminal acetyltransferases are associated with ribosomes. J Cell Biochem.

[16] Polevoda B, Arnesen T, Sherman F (2009). A synopsis of eukaryotic Nalpha-terminal acetyltransferases: nomenclature, subunits and substrates. BMC Proc.

[17] Aksnes H, Van Damme P, Goris M, Starheim KK, Marie M, Stove SI (2015). An organellar nalpha-acetyltransferase, naa60, acetylates cytosolic N termini of transmembrane proteins and maintains Golgi integrity. Cell Rep.

[18] Kalvik TV, Arnesen T (2013). Protein N-terminal acetyltransferases in cancer. Oncogene.

[19] Aksnes H, Hole K, Arnesen T (2015). Molecular, cellular, and physiological significance of N-terminal acetylation. Int Rev Cell Mol Biol.

[20] Yu M, Ma M, Huang C, Yang H, Lai J, Yan S (2009). Correlation of expression of human arrest-defective-1 (hARD1) protein with breast cancer. Cancer Invest.

[21] Lee CF, Ou DSC, Lee SB, Chang LH, Lin RK, Li YS (2010). hNaa10p contributes to tumorigenesis by facilitating DNMT1-mediated tumor suppressor gene silencing. J Clin Invest.

[22] Ren T, Jiang B, Jin G, Li J, Dong B, Zhang J (2008). Generation of novel monoclonal antibodies and their application for detecting ARD1 expression in colorectal cancer. Cancer Lett.

[23] Hua KT, Tan CT, Johansson G, Lee JM, Yang PW, Lu HY (2011). *N*-Alpha-acetyltransferase 10 protein suppresses cancer cell metastasis by binding PIX proteins and inhibiting Cdc42/Rac1 activity. Cancer Cell.

[24] Ametzazurra A, Larrea E, Civeira MP, Prieto J, Aldabe R (2008). Implication of human *N*-alpha-acetyltransferase 5 in cellular proliferation and carcinogenesis. Oncogene.

[25] Starheim KK, Arnesen T, Gromyko D, Ryningen A, Varhaug JE, Lillehaug JR (2008). Identification of the human *N*(alpha)-acetyltransferase complex B (hNatB): a complex important for cell-cycle progression. Biochem J.

[26] Starheim KK, Gromyko D, Evjenth R, Ryningen A, Varhaug JE, Lillehaug JR (2009). Knockdown of human N alpha-terminal acetyltransferase complex C leads to p53-dependent apoptosis and aberrant human Arl8b localization. Mol Cell Biol.

[27] Starheim KK, Gevaert K, Arnesen T (2012). Protein N-terminal acetyltransferases: when the start matters. Trends Biochem Sci.

[28] Song OK, Wang X, Waterborg JH, Sternglanz R (2003). An Nalpha-acetyltransferase responsible for acetylation of the N-terminal residues of histones H4 and H2A. J Biol Chem.

[29] Polevoda B, Hoskins J, Sherman F (2009). Properties of Nat4, an N(alpha)-acetyltransferase of *Saccharomyces cerevisiae* that modifies N termini of histones H2A and H4. Mol Cell Biol.

[30] Magin RS, Liszczak GP, Marmorstein R (2015). The molecular basis for histone H4- and H2A-specific amino-terminal acetylation by NatD. Structure.

[31] Van Damme P, Stove SI, Glomnes N, Gevaert K, Arnesen T (2014). A *Saccharomyces cerevisiae* model reveals in vivo functional impairment of the Ogden syndrome N-terminal acetyltransferase NAA10 Ser37Pro mutant. Mol Cell Proteomics.

[32] Hole K, Van Damme P, Dalva M, Aksnes H, Glomnes N, Varhaug JE (2011). The human *N*-alpha-acetyltransferase 40 (hNaa40p/hNatD) is conserved from yeast and N-terminally acetylates histones H2A and H4. PLoS ONE.

[33] Schiza V, Molina-Serrano D, Kyriakou D, Hadjiantoniou A, Kirmizis A (2013). N-alpha-terminal acetylation of histone H4 regulates arginine methylation and ribosomal DNA silencing. PLoS Genet.

[34] Riesen M, Morgan A (2009). Calorie restriction reduces rDNA recombination independently of rDNA silencing. Aging Cell.

[35] Smith DL, Li C, Matecic M, Maqani N, Bryk M, Smith JS (2009). Calorie restriction effects on silencing and recombination at the yeast rDNA. Aging Cell.

[36] Liu Y, Zhou D, Zhang F, Tu Y, Xia Y, Wang H (2012). Liver Patt1 deficiency protects male mice from age-associated but not high-fat diet-induced hepatic steatosis. J Lipid Res.

[37] The Human Atlas. http://www.proteinatlas.org/ENSG00000110583-NAA40/cancer. Accessed 5 Nov 2015

[38] Hanahan D, Weinberg RA (2011). Hallmarks of cancer: the next generation. Cell.

[39] Norbury CJ, Hickson ID (2001). Cellular responses to DNA damage. Annu Rev Pharmacol Toxicol.

[40] Parrish AB, Freel CD, Kornbluth S (2013). Cellular mechanisms controlling caspase activation and function. Cold Spring Harb Perspect Biol.

[41] Ashkenazi A, Dixit VM (1998). Death receptors: signaling and modulation. Science.

[42] McIlwain DR, Berger T, Mak TW (2013). Caspase functions in cell death and disease. Cold Spring Harb Perspect Biol.

[43] Oltvai ZN, Milliman CL, Korsmeyer SJ (1993). Bcl-2 heterodimerizes in vivo with a conserved homolog, Bax, that accelerates programmed cell death. Cell.

[44] Brentnall M, Rodriguez-Menocal L, De Guevara RL, Cepero E, Boise LH (2013). Caspase-9, caspase-3 and caspase-7 have distinct roles during intrinsic apoptosis. BMC Cell Biol.

[45] Lopez J, Tait SW (2015). Mitochondrial apoptosis: killing cancer using the enemy within. Br J Cancer.

[46] Koff JL, Ramachandiran S, Bernal-Mizrachi L (2015). A time to kill: targeting apoptosis in cancer. Int J Mol Sci.

[47] Bunz F, Dutriaux A, Lengauer C, Waldman T, Zhou S, Brown JP (1998). Requirement for p53 and p21 to sustain G2 arrest after DNA damage. Science.

[48] Harasawa R, Mizusawa H, Nozawa K, Nakagawa T, Asada K, Kato I (1993). Detection and tentative identification of dominant mycoplasma species in cell cultures by restriction analysis of the 16S-23S rRNA intergenic spacer regions. Res Microbiol.

[49] Liu Z, Liu Y, Wang H, Ge X, Jin Q, Ding G (2009). Patt1, a novel protein acetyltransferase that is highly expressed in liver and downregulated in hepatocellular carcinoma, enhances apoptosis of hepatoma cells. Int J Biochem Cell Biol.

[50] Wu D, Chen X, Guo D, Hong Q, Fu B, Ding R (2005). Knockdown of fibronectin induces mitochondria-dependent apoptosis in rat mesangial cells. J Am Soc Nephrol.

[51] Fornaro M, Plescia J, Chheang S, Tallini G, Zhu YM, King M (2003). Fibronectin protects prostate cancer cells from tumor necrosis factor-alpha-induced apoptosis via the AKT/survivin pathway. J Biol Chem.

[52] Elmore S (2007). Apoptosis: a review of programmed cell death. Toxicol Pathol.

[53] Sabbagh L, Kaech SM, Bourbonniere M, Woo M, Cohen LY, Haddad EK (2004). The selective increase in caspase-3 expression in effector but not memory T cells allows susceptibility to apoptosis. J Immunol.

[54] Droin N, Dubrez L, Eymin B, Renvoize C, Breard J, Dimanche-Boitrel MT (1998). Upregulation of CASP genes in human tumor cells undergoing etoposide-induced apoptosis. Oncogene.

[55] Druškovič M, Šuput D, Milisav I (2006). Overexpression of caspase-9 triggers its activation and apoptosis in vitro. Croat Med J.

[56] Ethier C, Labelle Y, Poirier GG (2007). PARP-1-induced cell death through inhibition of the MEK/ERK pathway in MNNG-treated HeLa cells. Apoptosis.

[57] Sousa FG, Matuo R, Soares DG, Escargueil AE, Henriques JA, Larsen AK (2012). PARPs and the DNA damage response. Carcinogenesis.

[58] Liu Y, Bodmer WF (2006). Analysis of P53 mutations and their expression in 56 colorectal cancer cell lines. Proc Natl Acad Sci USA.

[59] He X, Liao J, Liu F, Yan J, Yan J, Shang H (2015). Functional repair of p53 mutation in colorectal cancer cells using trans-splicing. Oncotarget.

[60] Drygin D, Lin A, Bliesath J, Ho CB, O’Brien SE, Proffitt C (2011). Targeting RNA polymerase I with an oral small molecule CX-5461 inhibits ribosomal RNA synthesis and solid tumor growth. Cancer Res.

[61] Gromyko D, Arnesen T, Ryningen A, Varhaug JE, Lillehaug JR (2010). Depletion of the human Nalpha-terminal acetyltransferase A induces p53-dependent apoptosis and p53-independent growth inhibition. Int J Cancer.

[62] Arnesen T, Gromyko D, Pendino F, Ryningen A, Varhaug JE, Lillehaug JR (2006). Induction of apoptosis in human cells by RNAi-mediated knockdown of hARD1 and NATH, components of the protein *N*-alpha-acetyltransferase complex. Oncogene.

[63] Polevoda B, Norbeck J, Takakura H, Blomberg A, Sherman F (1999). Identification and specificities of N-terminal acetyltransferases from *Saccharomyces cerevisiae*. EMBO J.

[64] Tweedie-Cullen RY, Brunner AM, Grossmann J, Mohanna S, Sichau D, Nanni P (2012). Identification of combinatorial patterns of post-translational modifications on individual histones in the mouse brain. PLoS ONE.

[65] Foyn H, Jones JE, Lewallen D, Narawane R, Varhaug JE, Thompson PR (2013). Design, synthesis, and kinetic characterization of protein N-terminal acetyltransferase inhibitors. ACS Chem Biol.

[66] Sarosiek KA, Ni Chonghaile T, Letai A (2013). Mitochondria: gatekeepers of response to chemotherapy. Trends Cell Biol.

