# Supplementary material for: Depletion of histone N-terminal-acetyltransferase Naa40 induces p53-independent apoptosis in colorectal cancer cells via the mitochondrial pathway
Source: Apoptosis. 2015 Dec 14;21:298–311. doi: 10.1007/s10495-015-1207-0 (PMC4746217; doi:10.1007/s10495-015-1207-0)
Supplement: Supplementary file 1 — Supplementary material 1 (DOCX 2223 kb) [file 10495_2015_1207_MOESM1_ESM.docx]

| Gene | Forward 5’🡪3’ | Reverse 5’🡪3’ |
| --- | --- | --- |
| Naa40 | TGGTGCCTACCAGTTCTTCA | CTCCGGCTCAGGATCTCATA |
| β-actin | GGCATCCTCACCCTGAAGTA | AGGTGTGGTGCCACATTTTC |
| Caspase-9 | cgacatctttgagcagtggg | ccctttcaccgaaacagcat |
| Caspase-8 | ACTTTGCCAGAGCCTGAGAG | GCATCTGTTTCCCCATGTTT |
| Caspase-7 | gaatgacagagttgccaggc | tccttggtgagcatggagac |
| Caspase-6 | tggagctgacttcctcatgt | accagtgtgaggagttctgt |
| Caspase-3 | atgcacattcttacccgggt | gcgtcaaaggaaaaggactca |
| PARP-1 | ggcaaaactacccctgatcc | cacaccagatgaaatcccgg |
| 5.8S rRNA | actcggctcgtgcgtc | gcgacgctcagacagg |
| 28S rRNA | agaggtaaacgggtggggtc | ggggtcgggaggaacgg |
| 18S rRNA | GCAAATTACCCACTCCCGAC | CCCTCCAATGGATCCTCGTT |
| 45S rRNA | gaacggtggtgtgtcgtt | gcgtctcgtctcgtctcact |

**Supplementary Data 1**: Sequence of primers used in RT-PCR.


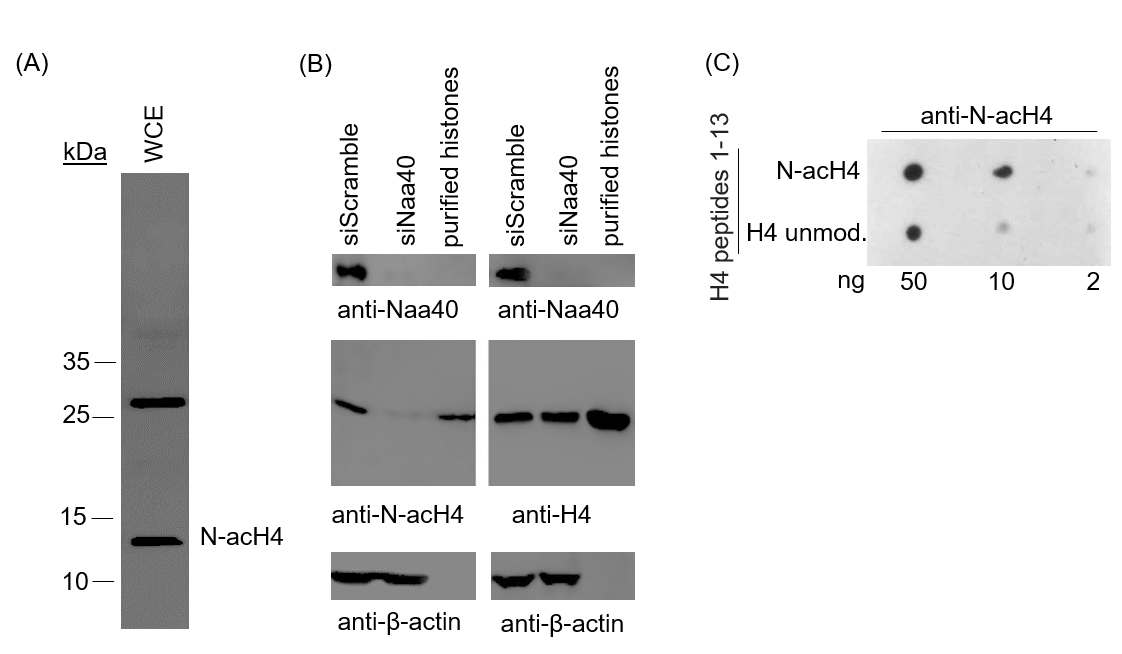


**Supplementary data 2**: N-acH4 antibody specifically recognises the N-terminally acetylated version of histone H4. **(A)** Immunoblot analysis of whole cell extracts (WCE) prepared from HCT116 cells using the antibody against N-acH4. **(B)** Immunoblot analysis of siScramble and siNaa40-treated cells as well as purified histones extracted from calf thymus, using the antibodies against Naa40, N-acH4, total H4 and β-actin. **(C)** Dot blot analysis using the N-acH4 antibody against H4 N-terminally acetylated (N-acH4) and unmodified (H4 unmod) peptides representing the first 13 amino acids of H4 (SGRGKGGKGLGKC). The amount of loaded peptides in nanograms (ng) is shown below the blot.


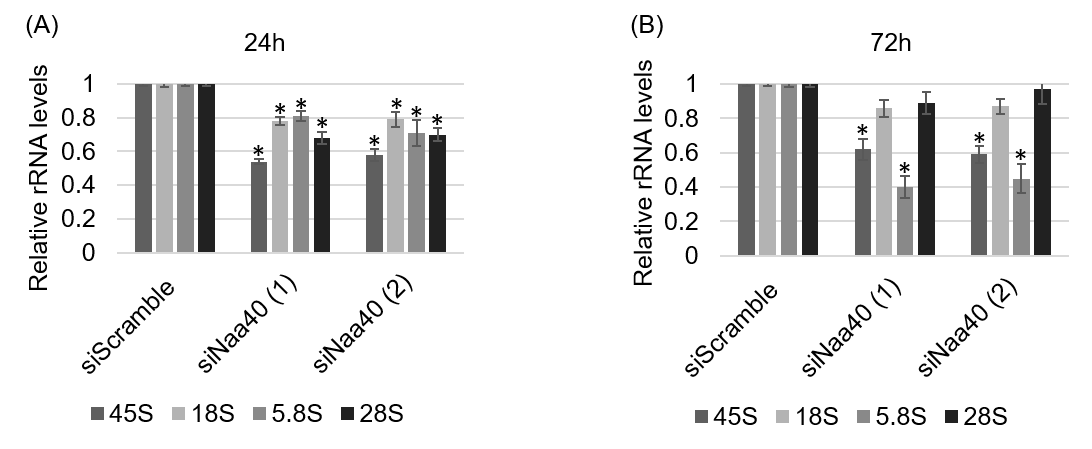


**Supplementary data 3**: Depletion of Naa40 reduces rRNA expression. Expression levels of rRNAs 45S, 18S, 5.8S and 28S were analyzed by qRT-PCR using total RNA extracted from siScramble and siNaa40-treated samples, **(A)** 24h and **(B)** 72h post-transfection. The levels of rRNA were quantified and then normalised to the levels of β-actin mRNA, whose expression remained unchanged. The data represent the mean of three replicates and are representative of three independent experiments ±S.D (*p* value <0.02).


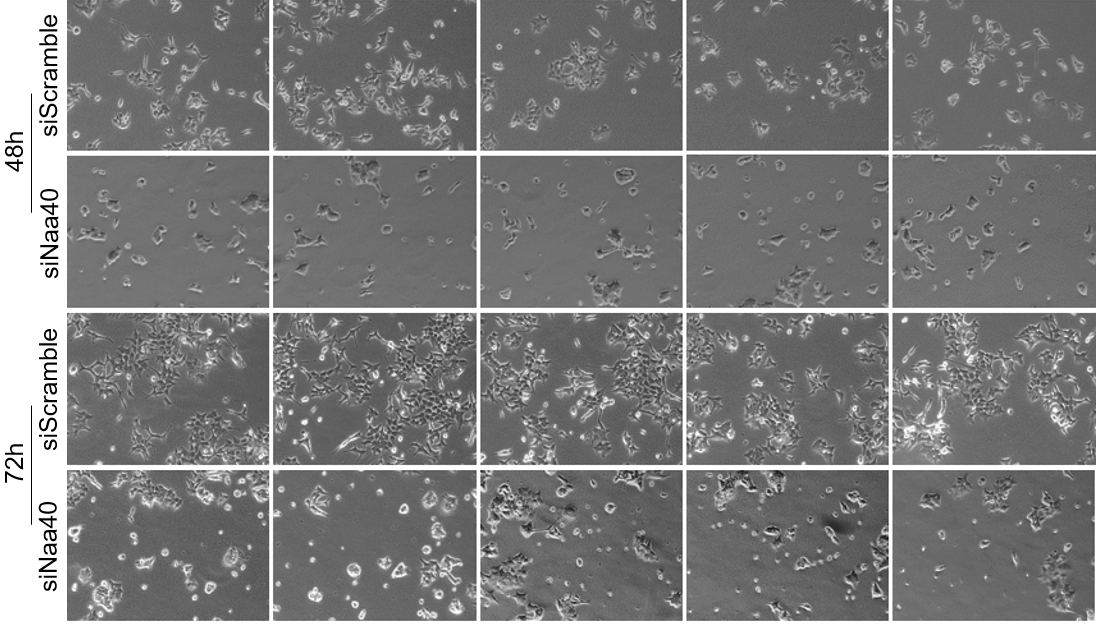


**Supplementary data 4**: Naa40-kncokdown reduces the viability of HCT116 cells. Phase contrast microscopy images of HCT116 cells shown at different time points (48h and 72h) after treatment with siScramble or siNaa40. The images are representative fields of view from at least three reproducible independent experiments.


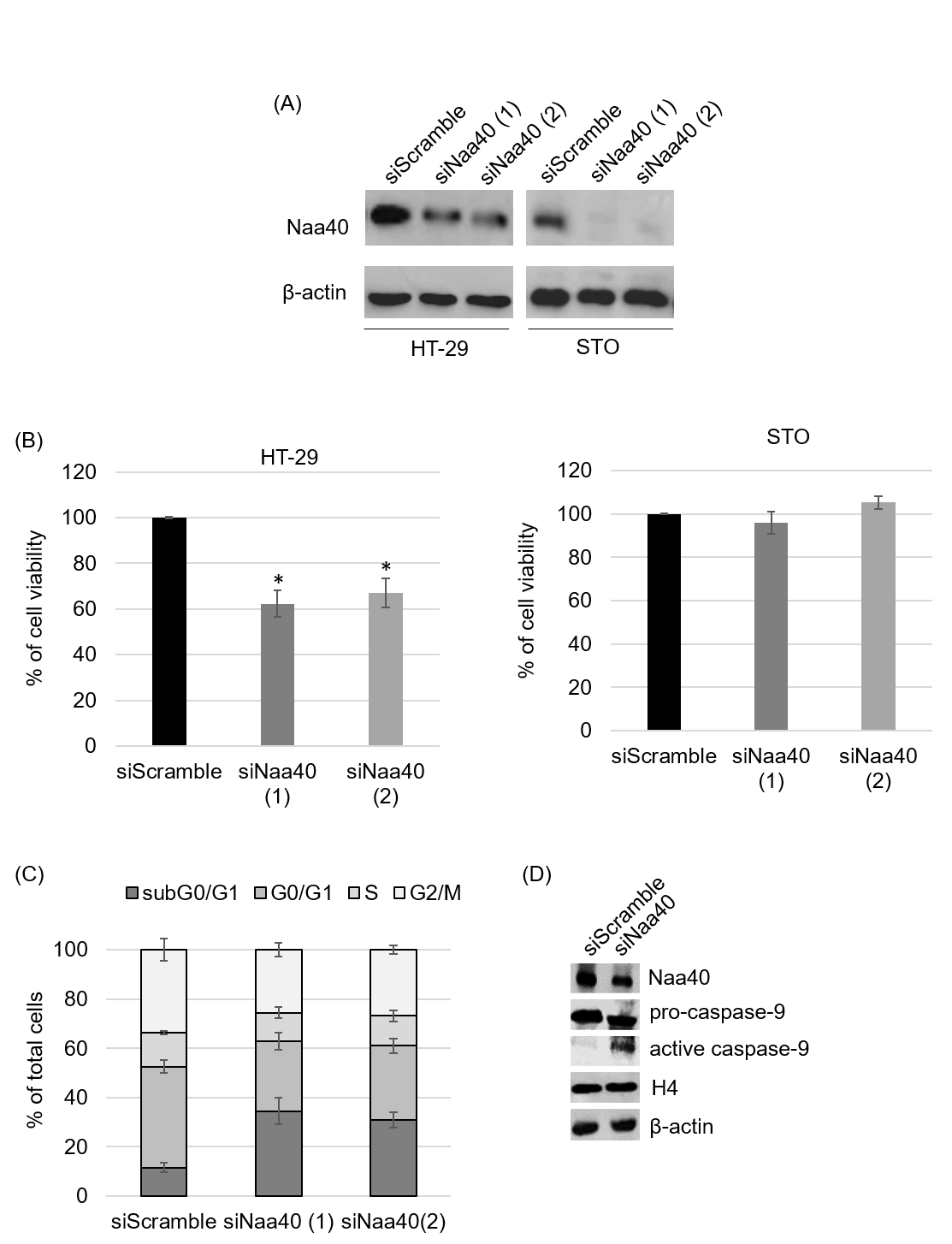


**Supplementary data 5**: Depletion of Naa40 induces apoptosis in HT-29 colon cancer cells but not in STO mouse embryonic fibroblasts. **(A)** Immunoblot analysis of HT-29 and STO cells that were transfected with 25nM and 7.5nm, respectively, of Naa40 siRNA-1, Naa40 siRNA-2 or scramble siRNA and incubated 72h. Antibodies against Naa40 and β-actin, as a loading control, were used. **(B)** MTT cell viability assay. Cell viability of HT-29 cells (*left panel*) and STO cells (*right panel*) is shown as a percentage relative to the scramble-siRNA control. Statistically significant changes (*p* value <0.02) are indicated with an *asterisk* (*) **(C)** Cell-cycle analysis of HT-29 cells after treatment with a scramble-siRNA or Naa40-siRNAs, for 72h. **(D)** Immunoblot analysis of siScramble and siNaa40-treated cells (72h) using antibodies against Naa40, caspase-9, total H4 and β-actin that was used as a loading control. The data in (B) and (C) represent the mean of three independent experiments ±S.D *(p* value <0.02).

**
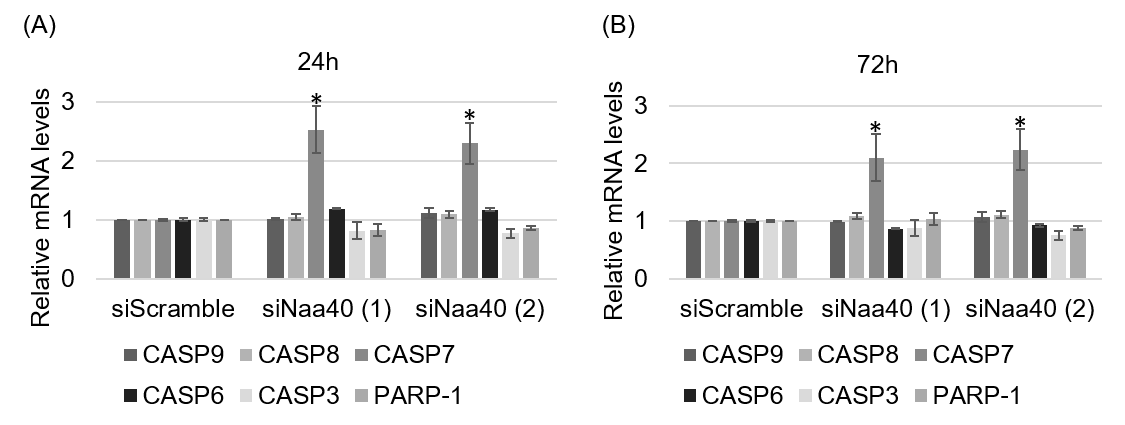
**

**Supplementary data 6**: Effect of Naa40 depletion on the mRNA levels of pro-caspases and PARP-1. RT-PCR analysis of the mRNA levels of caspase-9, -8, -7, -6, -3 and PARP-1, 24h and 72h post-transfection. The results are normalised to the levels of β-actin mRNA. The data represent the mean of three replicates and are representative of three independent experiments ±S.D (*p* value <0.02).


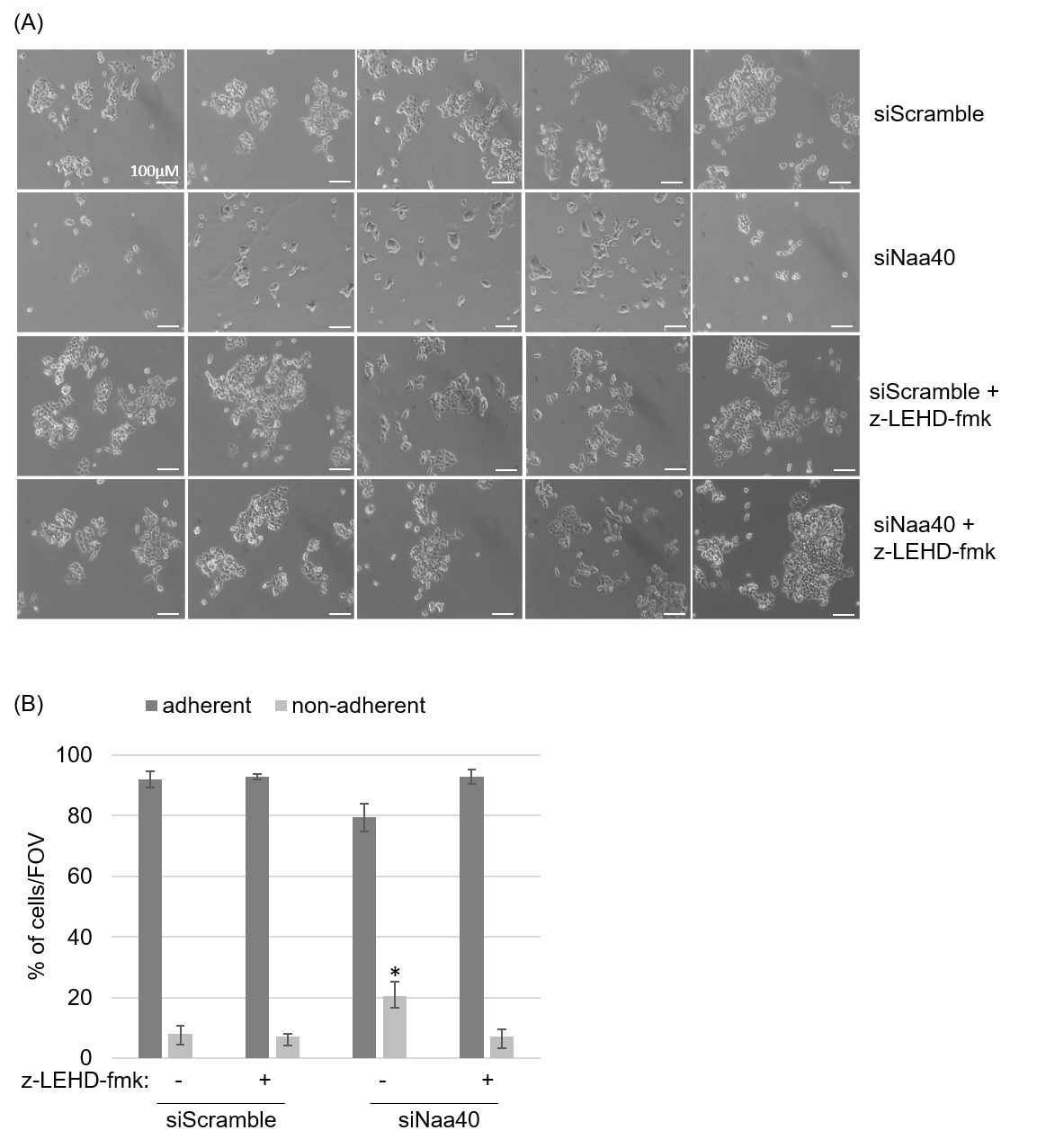


**Supplementary data 7**: Caspase-9 inhibitor prevents loss of cell viability in Naa40-depleted HCT116 cells. **(A)** Phase contrast microscopy images, 48h post-Naa40 knockdown. HCT116 cells are transfected with siScramble or siNaa40 in the absence or presence of caspase-9 inhibitor z-LEHD-fmk. The images are representative fields of view from at least three reproducible independent experiments. **(B)** Quantitation of adherent and non-adherent cells per field of view, in the conditions mentioned in (A). Data represent the mean number of 15 fields of view taken from three independent experiments ±S.D (*p* value <0.02)


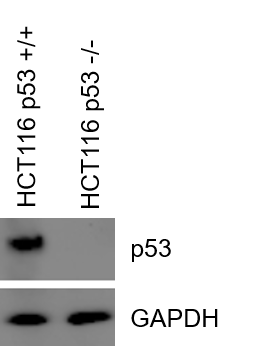


**Supplementary data 8**: Immunoblot analysis of HCT116 p53+/+ and HCT116 p53-/- whole cell extracts against p53 protein. GAPDH was used as a loading control.
